# Immune escape and resistance to immunotherapy in mismatch repair deficient tumors

**DOI:** 10.3389/fimmu.2023.1210164

**Published:** 2023-07-10

**Authors:** Guillaume Mestrallet, Matthew Brown, Cansu Cimen Bozkus, Nina Bhardwaj

**Affiliations:** ^1^ Division of Hematology and Oncology, Hess Center for Science & Medicine, Tisch Cancer Institute, Icahn School of Medicine at Mount Sinai, New York, NY, United States; ^2^ Extramural member, Parker Institute for Cancer Immunotherapy, San Francisco, CA, United States

**Keywords:** MMRd, MSI-H, microsatellite unstable (high), colorectal cancer, PD1 (programmed cell death protein 1), resistance, myeloid cells, immunotherapy

## Abstract

Up to 30% of colorectal, endometrial and gastric cancers have a deficiency in mismatch repair (MMR) protein expression due to either germline or epigenetic inactivation. Patients with Lynch Syndrome who inherit an inactive MMR allele have an up to 80% risk for developing a mismatch repair deficient (MMRd) cancer. Due to an inability to repair DNA, MMRd tumors present with genomic instability in microsatellite regions (MS). Tumors with high MS instability (MSI-H) are characterized by an increased frequency of insertion/deletions (indels) that can encode novel neoantigens if they occur in coding regions. The high tumor antigen burden for MMRd cancers is accompanied by an inflamed tumor microenvironment (TME) that contributes to the clinical effectiveness of anti-PD-1 therapy in this patient population. However, between 40 and 70% of MMRd cancer patients do not respond to treatment with PD-1 blockade, suggesting that tumor-intrinsic and -extrinsic resistance mechanisms may affect the success of checkpoint blockade. Immune evasion mechanisms that occur during early tumorigenesis and persist through cancer development may provide a window into resistance pathways that limit the effectiveness of anti-PD-1 therapy. Here, we review the mechanisms of immune escape in MMRd tumors during development and checkpoint blockade treatment, including T cell dysregulation and myeloid cell-mediated immunosuppression in the TME. Finally, we discuss the development of new therapeutic approaches to tackle resistance in MMRd tumors, including cancer vaccines, therapies targeting immunosuppressive myeloid programs, and immune checkpoint combination strategies.

## Introduction

**Figure 1:**
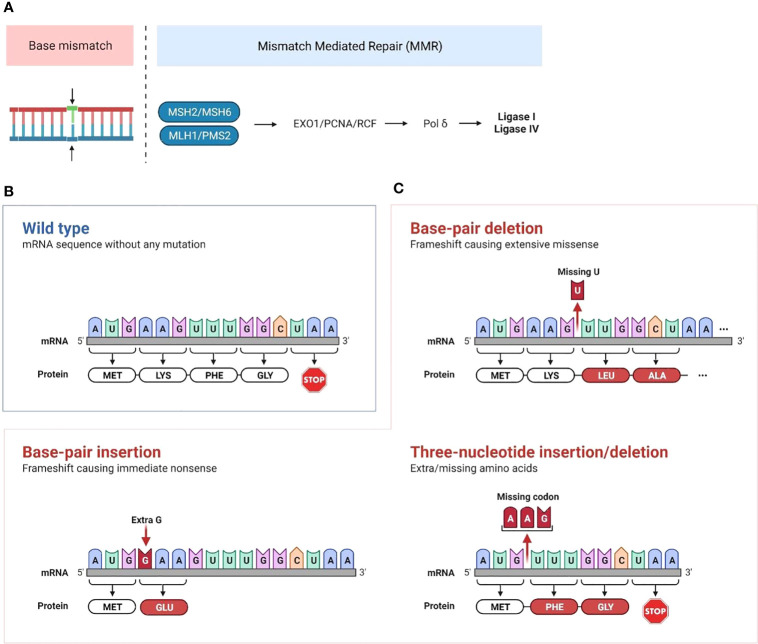
Mechanisms of mismatch repair deficiency leading to cancer development **(A)** Mismatch repair deficiency is characterized by mutations in or epigenetic inactivation of MMR genes (MLH1, MSH2, PMS2 and MSH6). Normally, proteins encoded by MMR genes detect errors in replication and recruit proteins encoded by MLH and PMS genes. This complex recruits the PCNA/exonuclease complex that excises the mismatch and repairs the DNA through insertion of correct bases. Then, Pol δ is recruited for both leading and lagging strand synthesis, and the ligases catalyze the joining of repaired DNA fragments. **(B, C)** Accumulation of mutations that are not repaired following MMR deficiency leads to microsatellite extension or shortening through base-pair or three-nucleotide insertion/deletion in microsatellite repeated sequences. This leads to the production of neoantigens if the altered transcript is a coding one. (Created with BioRender.com).

The mismatch repair (MMR) pathway plays a key role in repairing DNA damage. Proteins encoded by MMR genes detect errors in replication and recruit proteins encoded by MLH1, MSH2, MSH6 and PMS2 genes. MSH2 and MSH6 allow DNA mismatch/damage recognition. MLH1 and PMS2 are involved in the termination of mismatch-provoked excision. During DNA replication, the MMR complex recruits DNA exonuclease with PCNA to excise the mismatch. Then, Pol δ, RPA and PCNA promote DNA re-synthesis, and DNA ligase 1 promotes nick ligation (1) (**Figure 1A**).

MMR deficiency (MMRd) causes genomic instability and accumulation of DNA mutations. Patients who inherit inactivating mutations in mismatch repair genes have a significantly increased lifetime risk (up to 80%) for developing cancer, typically with an earlier onset. MMRd can occur in various cancer types, but is most frequently seen in colorectal, gastric, endometrial, and ovarian cancers (2–14).

MMRd can be induced by sporadic or inherited alterations in the MMR pathway proteins (15–17). The most common sporadic alteration is epigenetic silencing of MLH1, occurring in 80% to 95% of these cases (18). Multiple causes for epigenetic silencing of MLH1 leading to MSI-H cancers have been investigated. For example, protein phosphatase 2A (PP2A) inactivation induces microsatellite instability (MSI), neoantigen production and anti-tumor immunity. PP2A inactivation converts immunologically cold microsatellite-stable (MSS) tumors into MSI tumors through two different pathways: (a) by increasing retinoblastoma protein phosphorylation that leads to E2F and DNMT3A/3B expression with subsequent DNA methylation, resulting in MLH1 silencing, and (b) by increasing histone deacetylase (HDAC)2 phosphorylation that subsequently decreases the acetylation at the 9th lysine residue of the histone H3 protein (H3K9ac) and overall histone acetylation levels. It results in epigenetic silencing of MLH1 (19). Inherited alterations arise from germline loss-of-function mutations, which acquire mismatch repair deficiency following loss of heterozygosity, as observed in Lynch Syndrome (LS). LS is characterized by an inherited inactivating mutation at one allele of an MMR gene, with detection rates of 39% MLH1, 30% MSH2, 31% MSH6 (20–26). The germline prevalence of LS is as high as 1 in 320 individuals (27).

MMRd cancers typically display a phenotype of high microsatellite instability (MSI-H), which is characterized by exceptionally large somatic mutation rates, particularly insertions and deletions in microsatellite regions. Microsatellites are short, repeated DNA segments, typically 1 to 6 base pairs, which are scattered throughout the genome. Due to the repetitive nature of these sequence elements, microsatellite (MS) regions are prone to DNA replication errors. Accumulation of mutations that are not repaired following MMR deficiency leads to microsatellite extension or shortening (**Figures 1B, C**). When this occurs in coding regions of the genome, it leads to the production of single nucleotide variant (SNV) and frameshift peptide sequences that can potentially serve as novel immunogenic proteins that are uniquely expressed in cancer cells, called “neoantigens”. We and others have identified recurrent frameshift mutations in MSI-H cancers yielding highly immunogenic, shared frameshift-derived neoantigens that are targets of T cells in patients with MSI-H tumors. The high neoantigen loads in MSI-H cancers, combined with a favorable inflammatory signature in the TME, translate to a strong response to immune checkpoint blockade (ICB) (27, 28). However, approximately 50% of patients fail to respond to ICB treatment, suggesting that tumor-intrinsic and -extrinsic resistance mechanisms are likely affecting immune surveillance and the success of therapy (29, 30). Interestingly, phylogenetic trees of LS-associated colorectal cancer (CRC) tumor crypts indicate that MMR mutations contributing to MMR-deficiency are highly truncal and occur in nearly all clones within the lesion indicating that MMRd occurs early in tumor development (31). Despite MMRd presenting very early, tumor mutational burden and inflammatory microenvironment may not be sufficient to control tumor growth. MMRd pre-neoplastic lesions may progress to become malignant cancer cells by subsequent acquisition of tumor driver mutations in genes such as APC, KRAS, PI3K, PTEN, BRAF or p53 (27). Thus, studying immune responses in early/developing tumors will inform on resistance mechanisms in progressing tumors and in response to therapy.

In this review, we will discuss immune evasion mechanisms in developing MMRd/MSI-H tumors and following immune checkpoint blockade. Finally, we will discuss multiple immunotherapeutic strategies to overcome these evasion mechanisms in MSI-H cancers.

## Efficacy of immune checkpoint blockade in MSI-H tumors

**Figure 2:**
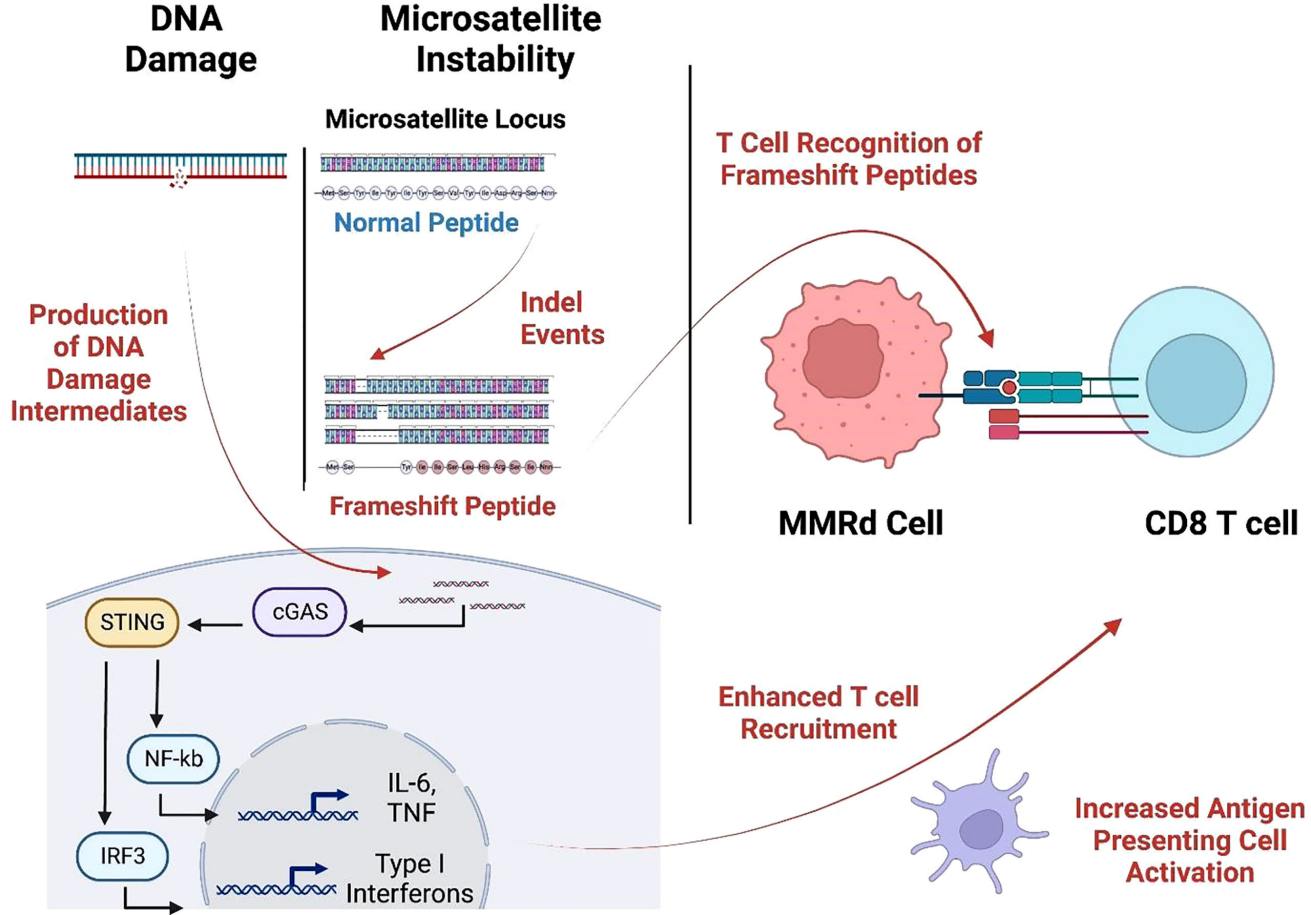
MMRd triggers T cell response through neoantigen production and the cGAS-STING pathway. Cytosolic DNA is sensed by the cGAS-STING pathways causing increased expression of IL-6, TNF, and type-I interferon which augments APC activation and T cell recruitment. These infiltrating T cells recognize frameshift-neoantigens encoded by insertion/deletion events at microsatellite loci. (Created with BioRender.com).

MSI-H cancers have exceptional response rates to ICB (around 50%). This recognition resulted in FDA approval for the tissue-agnostic deployment of checkpoint inhibitors in MSI-H or MMRd cancers (32). This favorable MMRd tumor response to ICB is probably due to high immune infiltration in the tumor microenvironment (TME), likely driven by multiple mechanisms, including a high neoantigen load and the activation of the cGAS-cGAMP-STING pathway (33–35) (**Figure 2**). We and others (27, 28) have recently identified highly immunogenic, shared frameshift-derived neoantigens that are targets of T cells in patients with MSI-H tumors and are associated with T cell infiltration into the tumor bed (27). Moreover, the expression of unstable DNA intermediates in MMRd tumor cells activates cGAS-cGAMP-STING signaling inducing the production of type I interferons, IL-6 and TNF. These in turn activate innate and adaptive immune responses by increasing T cell and antigen presenting cell (APC) recruitment into TME, enhanced antigen uptake and presentation, and activation of T cells by DCs in draining lymph nodes. Together, this inflammatory and antigen target rich environment results in more effective anti-tumor immune responses, thereby presenting an opportunity for successful ICB treatment in MSI-H cancer patients (36).

In 2015, a small phase 2 study showed that MMRd status predicted clinical benefit of ICB with pembrolizumab (anti-PD-1) (33). The response and progression-free survival rates were 40% and 78% respectively for MMRd CRC, and 0% and 11% for MMRp CRC, respectively. Patients with MMRd non-CRC (small bowel, gastric, endometrial, ampullary or cholangiocarcinoma) had responses similar to MMRd CRC patients (response rate, 71%; progression-free survival rate, 67%). In 2017, a larger phase 2 study of 12 different tumor types further confirmed that tumor control by ICB in MMRd cancers is not just limited to CRC (34). Objective radiographic responses were observed in 53% of patients, and complete responses were achieved in 21% of MMRd CRC and non-CRC patients after 1 year. For subjects that received nivolumab (anti-PD-1) plus ipilimumab (anti-CTLA4), the investigator-assessed ORR was 55% amongst MMRd CRC patients (37). In 2020, a phase 3 study showed that pembrolizumab led to significantly longer progression-free survival than chemotherapy (5-fluorouracil–based therapy with or without bevacizumab or cetuximab) when received as first-line therapy for MMRd metastatic CRC (35). An overall response (complete or partial response), as evaluated with Response Evaluation Criteria in Solid Tumors (RECIST), was observed in 43.8% of the patients in the pembrolizumab group and 33.1% in the chemotherapy group. However, the observed increase in overall survival was not statistically significant. The safety profile was more favorable with pembrolizumab, with 22% of grade 3 and 4 adverse events attributable to pembrolizumab versus 66% with chemotherapy. In the United States, frontline pembrolizumab was approved for MSI-H CRC in 2017, and second-line nivolumab and ipilimumab was approved in 2018 for those who fail to respond to chemotherapy/targeted treatment. In Europe, frontline pembrolizumab and the combination of second-line nivolumab and ipilimumab were approved in 2021 (38). In accordance with the hypothesis that favorable MMRd tumor response to ICB is probably due to high tumor mutational burden (TMB) and the consequent high immune infiltration in the TME, ICB has also demonstrated significant clinical benefit in other inflamed/TMB-high tumors, such as melanoma and lung tumors (39–41).

Currently, there are several ongoing trials investigating anti-PD1/PD-L1 therapy for MSI-H CRC in both adjuvant and neoadjuvant settings. In the adjuvant setting, one ongoing trial (Alliance A021502, NCT02912559), is evaluating the role of PD-L1 blockade in patients with stage 3 MSI-H CRC, using mFOLFOX6 with or without atezolizumab. Another phase 3 trial (NCT04008030) is evaluating the role of PD-1 blockade with or without CTLA-4 blockade vs chemotherapy for patients with MSI-H CRC (42). Importantly in the neoadjuvant settings, ICB has led to remarkable results for patients with MSI-H CRC, with pathologic complete response rates ranging from 60% to 100% (38, 43). In a recent neoadjuvant trial, dostarlimab (anti-PD-1, already approved in metastatic disease) showed promising results in 12 patients with MMRd stage II or III rectal adenocarcinoma (44) where treatment response was assessed prior to initiation of chemoradiotherapy and surgery. The patients received a dose of 500 mg every 3 weeks for 6 months. 100% of the 12 patients had a clinical complete response, with no evidence of tumor on magnetic resonance imaging, positron emission tomography, endoscopic evaluation, digital rectal examination, or biopsy. No patients needed to proceed with chemoradiotherapy or undergo surgery. Other trials evaluated extending the use of upfront PD-1 blockers (dostarlimab, toripalimab, sintilimab) to 6 months in MSI-H localized rectal cancer leading to complete clinical responses in 100%, 65-88% and 75% of patients, respectively, allowing a non-surgical approach in this subset (43–45). In summary, ICB has been shown to provide a significant clinical benefit as frontline, beyond frontline and in neoadjuvant settings for MMRd tumors (**Table 1**).

**Table 1:** Selected ICB trials demonstrating high efficacy for MMRd CRC and other TMB-high tumors.

Drug/phase	Cancer	Response	Ref
Pembrolizumab small phase 2 (2015)	MMRd CRC	Response rate: 40% Progression free survival rate: 78%	(33)
	MMRp CRC	Response rate: 0% Progression free survival rate: 11%	(33)
	MMRd non-CRC	Response rate: 40% Progression free survival rate: 78%	(33)
Pembrolizumab phase 2 (2018)	MMRd CRC and non-CRC	Objective radiographic responses: 53% Complete responses: 21%	(34)
Nivolumab plus Ipilimumab phase 2 (2018)	MMRd CRC	Overall response rate: 55%	(37)
Pembrolizumab vs chemotherapy phase 3 (2020)	MMRd metastatic CRC	Complete or partial response: 43.8% vs 33.1% No difference in overall survival Grade 3 and 4 adverse events: 22% vs 66%	(35)
Dostarlimab neoadjuvant (2022)	MMRd stage II or III rectal adenocarcinoma	Complete response: 100% (only 12 patients)	(44)
Ipilimumab plus Pembrolizumab or Nivolumab vs Ipilimumab (2021)	Metastatic melanoma	Objective response rate: 31% vs 13% Median overall survival: 20.4 months vs 8.8 months	(40)
Pembrolizumab vs chemotherapy (2019)	NSCLC	Overall response rate: 45% vs 28%	(41)

However, on average 50% or more of patients with advanced MMRd stage IV CRC fail to respond to ICB treatment, suggesting that tumor-intrinsic and -extrinsic resistance mechanisms are likely affecting immune surveillance and the success of therapy (29, 30). Therefore, it is critical to investigate the specific immune resistance mechanisms that develop in these patients during tumor evolution and following ICB failure. Better characterization of these resistance mechanisms of MSI-H tumors will be essential for identifying associated biomarkers and developing additional interventions that complement ICB to reduce tumor burden.

## Immune escape mechanisms in developing MSI-H tumors

**Figure 3:**
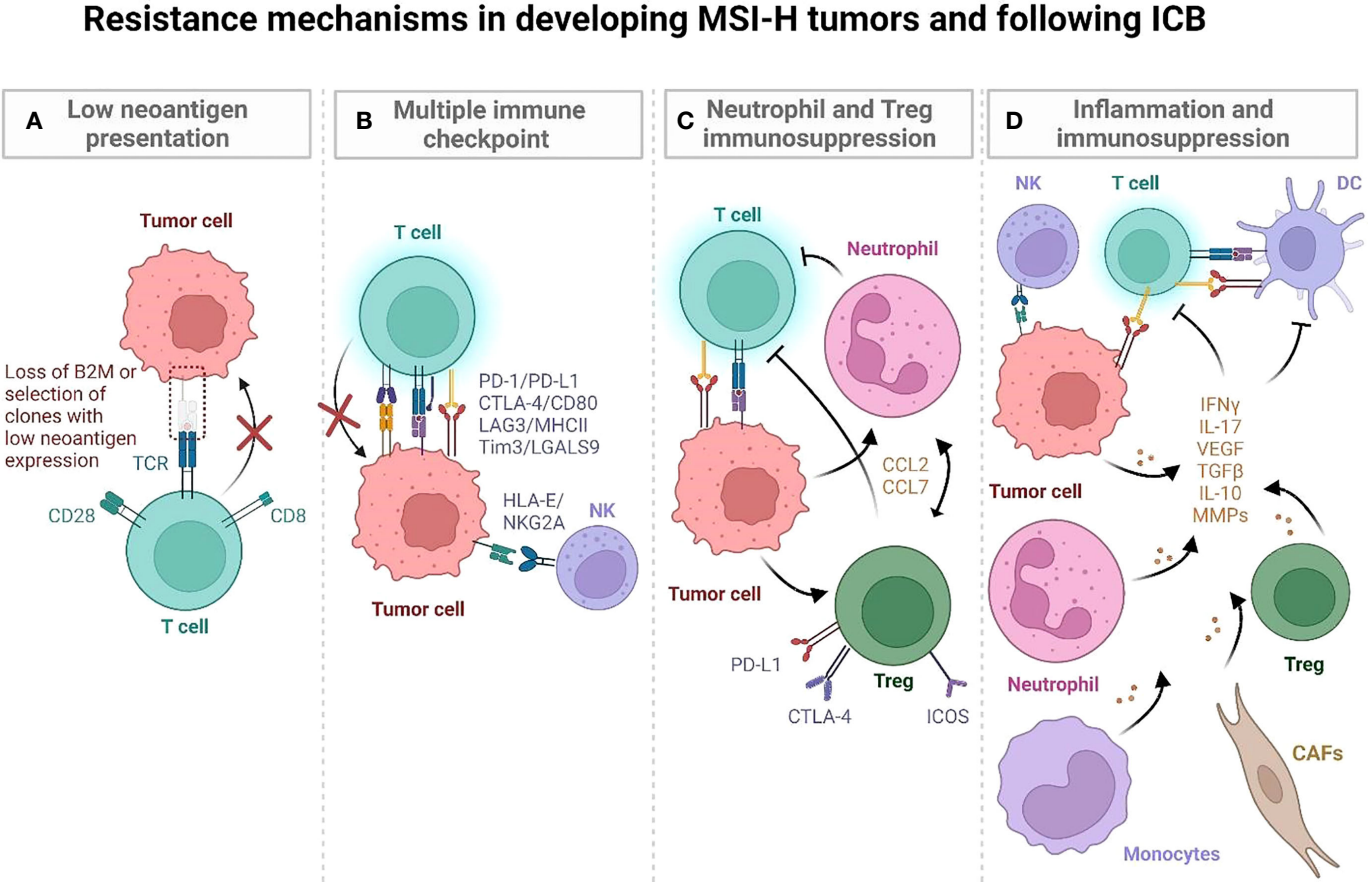
Postulated mechanisms by which MSI-H tumors evade immune recognition during development and following ICB. **(A)** LS-associated polyps initially have low mutational and neoantigen burden. Immunoediting of quality (immunogenic) neoantigens with low and/or subclonal expression and impaired antigen presentation may lead to immune evasion in developing tumors. **(B)** LS associated polyps display an immune activation profile characterized by CD4 T cells, proinflammatory cytokines (TNF, IL-12), and CTLA-4, LAG3 and PD-L1 checkpoints. They progressively develop additional mutations and high TMB, which is characterized by T cells entering an exhaustion state. It may also involve an IFN-induced expression of HLA-E that inhibits CD8+ T cells and NK cells *via* the NKG2A/CD94 receptor. **(C, D)** MSI-H tumors can develop strong anti-tumor immune responses as well as developing immune suppressive inflammatory hubs that compromise anti-tumor immunity (47). In human MSI-H tumors, an inflammatory hub populated by inflammatory Tregs **(C)**, MMP-expressing CAFs **(D)**, monocytes, and neutrophils may further increase immune escape during MSI-H tumor development, promoting tumor angiogenesis and tissue remodeling (47). (Created with BioRender.com).

Genomic and transcriptomic characterization have shown that MSI-H CRCs are immunologically heterogeneous despite having comparable TMB (46). Most are highly infiltrated by immune cells, but there are also MSI-H CRCs with low immune infiltration that are associated with mucinous histology, KRAS mutations and Wnt/Notch activation, greater tumor size, distant metastasis or early recurrence. Comparison of immune-high and -low MSI-H CRCs revealed differential mutational and immune gene signature enrichments, suggesting differences in clonal evolution and immune escape mechanisms of tumors (**Figure 3A**) (46). Below we characterize the highly prevalent features of immune escape in developing MMRd tumors, specifically focusing on Lynch syndrome (LS) where it is possible to follow the trajectory of immune infiltration at different stages of tumor development as these patients regularly undergo cancer screening.

### Immune escape due to T cell dysregulation and multiple checkpoint expression

LS-associated polyps and normal epithelium generally have low neoantigen burden but display an immune activation profile that is characterized by infiltrating CD4 T cells, proinflammatory cytokines (TNF, IL-12), and LAG3 and PD-L1 checkpoints suggesting early immune activation (48) (**Figure 3B**). Interestingly, a smaller subset of MMRd LS polyps have been characterized by high mutation and neoantigen rates, comparable to hypermutant tumors. This subset of cells also expresses CTLA4 (48). These findings suggest that neoantigen presentation, immune activation, and subsequent immune surveillance/escape may occur early in tumorigenesis. Further supporting the early immune activation in LS, normal colonic mucosa specimens from cancer-free patients were shown to be highly infiltrated by immune cells, including CD45+, CD8+, natural killer (NK), dendritic, mast, and B-cell populations, at even greater levels than tumor-distant normal colonic mucosa of LS patients with CRC (49). Notably, the density of CD3+ T cells in cancer-free LS patients correlated with increased time to cancer onset, implicating T cells as potentially mediating cancer risk in normal mucosa. This indicates that productive T cell infiltration and surveillance in normal colonic epithelium may be critical for eliminating early MMRd lesions and that lower infiltration/dysfunctional T cell states may compromise this early anti-tumor immunity. Indeed, an enrichment of FOXP3-positive regulatory T cells (Tregs), an immunosuppressive population found in LS carcinomas, was observed in the normal mucosa of LS patients, both with and without CRC, compared to non-LS control specimens. In addition, CD8+ T cells within the normal mucosa of LS patients displayed an exhausted phenotype, together suggesting the early occurrence of immune regulatory mechanisms as possible factors in cancer progression (49) (**Figure 3C**).

Current ongoing work by our team is seeking to determine whether T cells in early LS recognize frameshift neoantigens in MMRd colonic crypts (50) and whether this neoantigen expression can precede the immune dysregulation observed in LS CRC patients. We hypothesize that chronic activation accompanying neoantigen recognition leads to T cell exhaustion (51) and concomitant infiltration of T regulatory cells (52) promoting the development of an immunosuppressive TME that compromises this early immune surveillance causing MMRd tumors to develop.

### Immune escape mediated by myeloid and pro-inflammatory stromal immunosuppressive programs

The TME is composed of various cell populations, including malignant cells, stromal fibroblasts, and adaptive and innate immune cells, generating a complex and heterogeneous ecosystem. It is likely that pro- and anti-tumorigenic processes in the TME are governed by multicellular interaction networks involving frequencies and spatial organization of cell subsets and inter- and intra-cellular feedback loops. To understand the cellular programs underlying immune response in CRC tumors, a recent study analyzed primary untreated tumors and adjacent normal tissue from MMRd and MMRp CRC patients (47). MMRd and MMRp tumors displayed shared inflammatory profiles characterized by reduced infiltration of specific anti-tumor effector cell populations and elevated immunosuppressive transcriptional programs within the TME compared to normal tissue. Examples of a deficiency of major effector populations involved in orchestrating and mediating tumor cell clearance included plasma cells, B cells, *IL7R*+ T cells and γδ-like T cells (47). MMRd and MMRp tumors were also enriched in Tregs, monocytes, and macrophages compared to normal colon (47). These monocytes and macrophages in luminal tumor hubs upregulate inflammation (*MMP12* and *MMP9*), myeloid cell recruitment (*CCL2* and *CCL7*), and tumor growth–promoting genes (*VEGF* and *EREG*) (47) (**Figure 3D**). Notably, compared to MMRp, MMRd tumors were marked by a greater expression of chemokines attracting monocytes and neutrophils, as well as *S100A8* and *S100A9* alarmins which may mediate immunosuppression by enhancing accumulation of myeloid-derived suppressor cells (MDSCs) with T cell inhibitory functions (53) and secretion of suppressive cytokines IL-10 and TGF-β (54). These data demonstrate that myeloid programs are altered in MMRd tumors, which likely contribute to immune escape during tumor progression.

Comparison of MMRd and MMRp CRC revealed that the main difference in the immune composition of tumors was in the T cell compartment (47). Consistent with the greater immunogenicity of MMRd tumors, MMRd T cells were enriched in programs involving cytotoxicity (granulysin, granzyme B, perforin) and exhaustion (PD-1, TOX, LAG3), suggesting chronic stimulation of tumor-reactive T cells. In some MMRd hubs, T cells producing IFN gamma formed foci with interferon-stimulated genes (ISG) and CXCR3L expressing myeloid cells providing an environment conducive to lymphocyte recruitment, leading to infiltration of MMRd tumors by *CXCL13*+ T cells (a marker suggestive of tumor antigen reactive T cells (49–52)) and *PDCD1* (PD-1)+ γδ-like T cells, which may increase the anti-tumor response. The presence of these inflammatory immunogenic hubs is therefore likely critical in the regulation of the T cell response and overall anti-tumor immunity in MMRd tumors.

However, persistent inflammatory signaling in MMRd tumors may also drive immunosuppression, such as IFN-mediated upregulation of immune checkpoints and recruitment of immunosuppressive stromal and myeloid cells (cancer-associated fibroblasts (CAFs), neutrophils, macrophages) through multiple cytokine gradients (CCL2, CCL7, VEGF, IL-10, TGFB, IL-17, G-CSF, GM-CSF, IL1B, CCL20) (47, 55–59). Notably, in MMRd tumors, ISG signatures were spatially correlated with expression of inhibitory molecules, *IDO1, PD-1, PD-L1, Tim3*. MMRd tumors were also enriched in inflammatory CAFs. These CAFs express matrix metalloproteinases (MMPs), known to contribute to tumor angiogenesis and tissue remodeling (60). Activated CAFs may secrete IL-17, which can further promote tumor expansion (61). Thus, myeloid and stromal cells may be implicated in suppression of anti-tumor responses and promotion of MMRd tumor growth.

Altogether, these findings suggest that subsets of MMRd tumors can develop strong anti-tumor immune responses, but they can also develop immunosuppressive inflammatory hubs that compromise anti-tumor immunity (47). However, the precise timing of when these immune evasion mechanisms occur remains to be understood. These studies also indicate novel target pathways that may be amenable to immune modulation, such as myeloid recruiting chemokines, immunosuppressive myeloid cell programs, and signaling by stromal cells. Further studies using animal models and patient specimens may help to better characterize the functionality of these pathways and the potential of myeloid and stromal targeting to overcome resistance.

## Immune escape mechanisms in MSI-H tumors following ICB

### Resistance to ICB mediated by insertion-deletion load and NK, CD8+ and γδ T cell infiltration

The accumulation of mutations in MMRd tumors increases the neoantigen load and can render tumors immunogenic and sensitive to programmed cell death-1 (PD-1) blockade (33–35). Yet, response to PD-1 blockade among MSI-H patients varies, and as many as half are refractory to treatment. Recently, it was shown that the degree of microsatellite instability (MSI intensity) (62) and resultant neoantigen load, in part, predict the variable responses to anti-PD-1 therapy in MMRd human and mouse tumors (28, 63–65). The extent of response is particularly associated with the accumulation of insertion-deletion (indel) mutations in MSI-H patients. Thus, MMRd tumor immune resistance may be explained by the selection of clones with low indel mutations.

Mechanisms underlying MSI-H phenotype may impact mutational loads and multicellular interaction networks in the TME, altering response to ICB treatment. A recent phase II clinical study compared response rates to pembrolizumab (anti-PD1) therapy in recurrent endometrial cancer (EC) patients with sporadic MMRd due to either mutational or epigenetic alterations (66, 67). MMRd cancers without hypermethylated MLH1 that have no detectable pathogenic germline MMR mutations are described to have Lynch-like Syndrome (LLS) (66). In this study the ORR to pembrolizumab was 100% in LLS patients but only 44% in epigenetic MMRd patients (66). The 3-year PFS and OS proportions were 100% versus 30% and 100% versus 43%, respectively, in LLS vs epigenetic MMRd. These striking differences in ICB response based on the molecular mechanism of MMRd could be due to the significantly higher TMB in mutational MMRd (LLS) than epigenetic MMRd. Thus, some neoantigens that were targeted by CD8+ T cells may be absent in a subset of MMRd tumors with epigenetic silencing of the MLH1 (**Figure 3A**). This may also be due to the transient epigenetic silencing of the MLH1 gene where a subset of tumor cells can retain MMR protein expression. Alternatively, inflammatory networks within the TME of LLS and epigenetic MMRd may differ, impacting ICB response. A follow-up study delineating the underlying mechanisms of ICB response in the same phase II trial revealed that effector CD8+ T cells were correlated with regression of mutational MMRd tumors (LLS), while it was activated CD16+ NK cells that were correlated in epigenetic MMRd (67). Therefore, the molecular mechanisms of MMRd may impact disease pathophysiology, altering tumor immune contexture, which could provide a mechanistic explanation for the variable therapeutic benefit of ICB across MSI-H cancers.

### Resistance to ICB mediated by defects in antigen presentation

A common mechanism of tumor immune escape is downregulation or loss of MHC class I molecules, which impairs CD8+ T cell-mediated control. MHC-I expression in tumor cells has been defined as a correlate of response to ICB (68). In MMRd cancers, expression of MHC-I alleles, as well as other components of antigen presentation machinery, such as beta 2 microglobulin (B2M) and TAP2, are commonly disrupted (69) (**Figure 3A**). However, despite an expected deficiency in antigen presentation, the majority of B2M-deficient MMRd CRC achieve clinical benefit from ICB therapy (70). One explanation is that B2M may be accessed from other cells by cell-to-cell contacts, rendering tumors class I-proficient. It can also be explained by the involvement of immune effector cells other than CD8+ T cells controlling these tumors. It was recently demonstrated that γδ T cells are key effectors of immunotherapy in MMRd cancers with MHC class I defects (71). ICB substantially increased the frequency of γδ T cells in B2M-deficient MMRd cancers, expressing high levels of PD-1, cytotoxic molecules, and killer-cell immunoglobulin-like receptors. Furthermore, NK and CD4+ T cells may also play important roles in targeting class-I deficient MMRd tumors. Human MMRd tumors expressing low levels of B2M display increased intratumoral CD4+ T cells (72). In murine models of B2M-/- MMRd tumors, T cell subset depletion experiments demonstrated that CD4+, but not CD8+, T cells were required for the efficacy of checkpoint blockade (anti-PD-1 + anti-CTLA-4) (72). However, the mechanisms of CD4+ T cell driven anti-tumor immunity in response to ICB in MHC-I-deficient MMRd cancers remain to be fully investigated.

### Resistance to ICB and immune cytolytic activity

A strong correlate of ICB response is intratumoral cytolytic activity, where reduced expression of cytolytic programs has been associated with resistance to anti-PD-1 therapy in multiple cancer types, including CRC (73–78). In immunotherapy naïve patients, RNAseq profiling has demonstrated an enrichment in cytolytic T cell-associated programs in MMRd endometrial tumors compared to MMRp tumors, such as an increase in tumor-reactive T cell populations highly expressing PD-1 and CD39 molecules (79) and T cells with high perforin (PRF1) expression (80). A single cell RNAseq study showed that in MMRd CRC tumors, subsets of T and NK cells acquire cytolytic properties (Granulysin (GNLY), Granzyme B (GZMB), and PRF1) at a greater degree than their MMRp counterparts (47). This has been further validated at the protein level by immunohistochemistry, where it was observed that MMRd CRC has significantly higher intratumoral GZMB expression than MMRp CRC (81). Supporting a role of cytolytic activity in ICB response, it was demonstrated that in MMRd tumors treated with anti-PD-1 immunotherapy, expression of cytolytic markers, e.g., GZMA and PRF1, were enriched in responders (82). Although CD8 T cells have been proven to be major contributors of tumor clearance following ICB treatment, GZMA and PRF1 expression in MSI-H tumors was also associated with high levels of activated memory CD4+ T cells, γδ T cells and macrophages (78). This indicates that, as would be expected, a coordinated cytolytic immune response from multiple cellular compartments is needed for the optimal tumor control and prolonged survival observed in these patients.

### Resistance to ICB mediated by Tregs and myeloid cell subsets

MSI-H tumors accumulate monocyte, granulocyte and neutrophil populations as noted earlier (47, 55) (**Figures 3C, D**). These populations may contain subsets with immunosuppressive features and may affect the ICB response by compromising the anti-tumor immunity and promoting cancer cell proliferation. Targeted deletion of PD-1 in granulocyte/macrophage progenitors (GMPs) induces or promotes anti-tumor immunity in multiple mouse tumor models (55). Those progenitors accumulate during tumor development and give rise to myeloid-derived suppressor cells (MDSCs), which mediate T cell dysfunction. Following PD-1 deletion, accumulation of GMP and MDSC was reduced leading to an increase of T effector memory cells. In PD-1-deficient GMPs, growth factors (G-CSF and GM-CSF) induced increased glycolysis, activity in the pentose phosphate pathway and TCA cycle and elevated cholesterol. Because cholesterol is required for differentiation of inflammatory macrophages and DC and promotes antigen-presenting function, it suggests that metabolic reprogramming and differentiation of effector myeloid cells might be a key mechanism of anti-tumor immunity mediated by ICB.

MSI-H tumors also accumulate Tregs (47). Resistance to ICB may involve Treg-mediated immunosuppression in conjunction with mast cells, as recently shown in a melanoma model (83). Reduced MHC-I expression and poor infiltration by CD8+, Granzyme B+ T cells were observed in tumor regions where Tregs and mast cells co-localize each other, with such features associated with resistance to anti-PD-1 treatment. Studies in 2D and 3D coculture models showed that human mast cells promote CRC growth *via* increases in cytokine production (CCL15 or SCF) and TLR2 stimulation (84). Indeed, TLR2 ligands, such as laminin-β1, HMGB1 and S100A9, are upregulated in CRC, and human mast cells express CCR1 and CCR3- surface receptors for CCL15. More studies are needed to characterize this mechanism driven by TLR2 and whether this pathway is upregulated in human MSI-H cancers resistant to anti-PD-1 therapy.

### Resistance to ICB mediated by IFN-signaling and inflammation

In humans, an IFNγ-induced signature, including CXCL9/CXCL13 expression, was associated with a favorable response to ICB in multiple tumor types (85–87). However, persistent ISG hubs in tumors, as shown in a recent MMRd CRC tumor atlas (47), may ultimately drive immunosuppression because of the negative feedback that upregulates immune checkpoints and co-inhibitory factors such as PD1/PDL1, Lag3/MHC-II, Tim3/LGALS9, and IDO1 through IFN overstimulation (47) (**Figure 3D**). Further supporting a negative regulatory role for IFN signaling, a recent study reported that epigenetically pre-encoded responsiveness to type I interferon in the peripheral immune system, assessed prior to therapy, defines the outcome of PD-1 blockade such that high responsiveness to IFN-I was associated with therapy failure (88). Since accumulation of DNA damage in MMRd cells may chronically stimulate IFN signaling *via* cGAS-STING pathway, it is important to fully decipher the role of IFN signaling to ICB response in MMRd patients.

IFN signaling can upregulate classical and non-classical MHC molecules. A recent study investigating immune evasion mechanisms by genome-scale *in vivo* CRISPR screens across cancer models treated with ICB (89) identified immune evasion genes and immunosuppressive checkpoints conserved across cancers, including the non-classical MHC class I molecule Qa-1b/HLA-E. Notably, loss of tumor IFNγ signaling sensitized many tumor models to ICB. Immunosuppression by tumor IFN sensing is mediated through two mechanisms. First, tumor upregulation of classical MHC class I inhibits natural killer cells. Second, IFN-induced expression of Qa-1b (functional homolog of HLA-E in mice) inhibits CD8+ T cells *via* the NKG2A/CD94 receptor, and NKG2A may be induced by ICB. Strong IFN signatures are associated with poor response to ICB in individuals with renal cell carcinoma or melanoma (89). As MSI-H tumors contain persistent inflammatory hubs, IFN-mediated upregulation of classical and non-classical MHC class I inhibitory checkpoints in these tumors may facilitate immune escape (47).

Resistance to ICB was also associated with inflammation due to obstructions, perforations, peritonitis, or other radiologically diagnosed inflammation in abdominal viscera or metastatic sites in patients with MMRd CRC (90). This may be due to the involvement of neutrophils *via* CD80/CD86-CTLA4 signaling which was demonstrated using MSI-H CRC patient-derived organoids (90). Immune resistance to ICB mediated by inflammation also involves multiple metabolic and canonical tumorigenic pathways. Specifically, gene set enrichment analysis demonstrated that MSI-H non-responders to ICB had upregulation of epithelial mesenchymal transition, angiogenesis, hypoxia, mTORC1, TNF-α, KRAS, Wnt/β-catenin and TGF-β signaling pathways (91). VEGF-A-associated pathways were enriched in non-responders, and accompanied by shorter progression-free survival and overall survival. Profiling of CRC clinical samples and preclinical studies also suggested that alterations in the Wnt and the JAK-STAT signaling pathways may be associated with ICB resistance (92, 93).

In summary, the mechanisms that mediate immune escape and resistance to immunotherapy in MMRd tumors are multifactorial. A complete understanding of these mechanisms in the ecological context of their TME as they occur throughout tumor development and progression is essential to develop novel targeted treatments that will improve the outcome of clinical interventions in MMRd cancers.

## Novel strategies to overcome immune resistance of MSI-H tumors

MSI-H tumors escape immune surveillance during their development and can become resistant to immunotherapy. Thus, it is crucial to develop strategies to overcome this resistance.

### Targeting T cell dysfunction and myeloid cell-mediated immunosuppression

**Figure 4:**
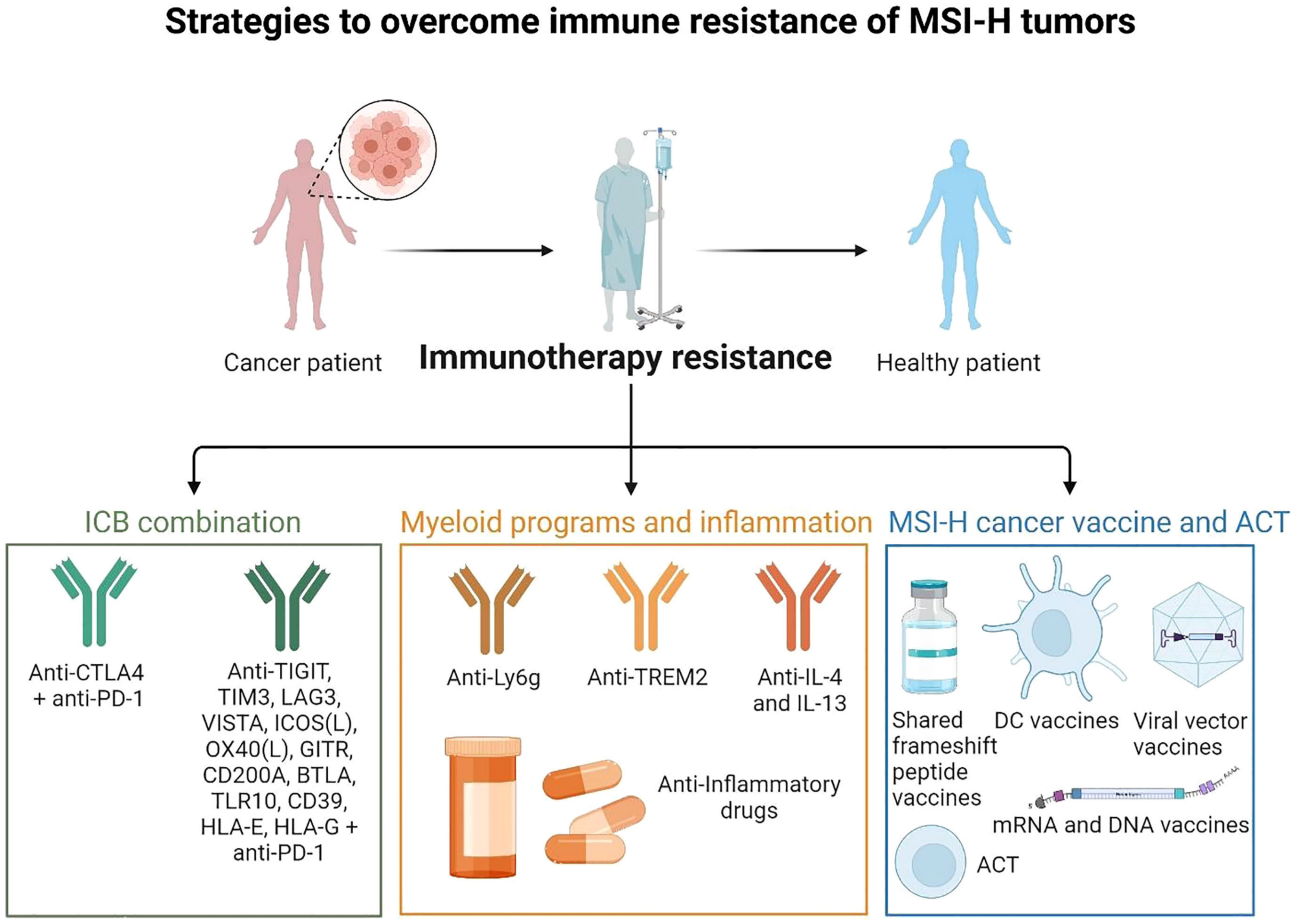
Strategies to overcome immune resistance of MSI-H tumors These strategies may include ICB combinations (anti-PD-1, anti-CTLA-4 to target Tregs, anti-LAG3, and others), targeting myeloid cells and inflammatory pathways and targeting myeloid immunosuppressive programs (anti-Ly6C to target neutrophils, anti-Trem2 to target macrophages, anti-IL-4 and IL-13 to target mregDCs, anti-inflammatory drugs to limit inflammatory hubs formation). It may act together with preventive strategies such as MSI-H cancer vaccines (shared frameshift peptide vaccines, ACT, DC vaccines, mRNA or viral vector vaccines). (Created with BioRender.com).

ICB combinations may serve as an alternative strategy to overcome immune resistance to checkpoint inhibitor monotherapies. LS-associated polyps and normal epithelium are infiltrated by T cells expressing LAG3, PD-L1 and CTLA-4 (48). Thus, targeting these checkpoints may restore anti-tumor immunity (**Figure 4**). It may also be of interest to target checkpoints described in other cancers, such as TIGIT (94–103). The HLA-E and HLA-G checkpoints, known to have immunosuppressive properties in epithelial tissue, can also be combined with PD-L1 (104–106). Indeed CRC tumors showing absence of MHC-I, HLA-E and HLA-G expression were related to a better overall survival, and it may be of interest to develop more blocking antibodies (such as monalizumab) and CAR T cells targeting these non-classical HLA, even if this might also affect non-tumor cells (107–110). Combination therapies may also enable clinicians to reduce the dosage of each monoclonal antibody, thereby minimizing the toxicity while maximizing the efficacy compared to ICB monotherapies.

Recently, it was shown that CD62L+ TPEX cells (precursors of exhausted T cells) are a stem-like population that is central to the responsiveness to anti-PD-1 therapy (111). In mice, TPEX cells co-express PD-1, the transcriptional regulators TCF1 and ID3 and the surface molecules CXCR5 and Ly108. These cells can continuously self-renew and give rise to functionally restrained effector T cells. On the contrary, exhausted effector T (TEX) cells co-express PD-1, TOX and TIM-3 but not TCF1, ID3, CXCR5 and Ly108. T cell exhaustion is an adaptation to continuous antigen stimulation in chronic infection and cancer. Although it protects against excessive immune-mediated tissue damage, it also contributes to viral or tumor persistence. The proliferative burst in response to PD-1 inhibition originates from CD62L+ TPEX cells and depends on MYB (111). MYB regulates two fundamental aspects of exhausted T cell responses: the downregulation of effector function and the long-term preservation of self-renewal capacity. Human MMRd cancer studies should investigate more the role of these cells following ICB. Future ICB therapies should better target this specific T cell subset in MSI-H tumors. New therapies might also target the CXCL13+ T cell subset that infiltrates MSI-H CRC (47). Indeed, a meta-analysis across 7 tumor types indicated that CXCL13 expression was a strong predictor of ICB response (86).

Targeting myeloid programs in combination with ICB may also help to overcome MSI-H tumor resistance. Patients with recurrent EC treated with immunotherapy with a pre-treatment neutrophil-to-lymphocyte ratio < 6 had a better overall survival (112). Studies in a MSI-H mouse model also showed that tumor-induced neutrophils countered anti-PD-1 efficacy (100). Since anti-CTLA-4 could restrict tumor-induced neutrophil accumulation, an anti-PD-1 + anti-CTLA-4 combination overcomes anti-PD-1 resistance in hosts with MSI-H tumors displaying abnormal neutrophil infiltration. Despite toxicity, Nivolumab (anti-PD-1) + ipilimumab (anti-CTLA-4) is a promising new treatment option for patients with MMRd/MSI-H CRC, with high response rates, encouraging progression-free survival and overall survival at 12 months (37).

In addition to neutrophils, new strategies may also target myeloid immunosuppressive pathways in MSI-H tumors. In murine models of sarcoma and CRC, TREM2 deficiency or TREM2 blockade in combination with PD-Ll blockade contributed to increased CD8 T cell activation and further limited tumor growth (113–115). Trem2 is expressed on macrophages and DCs (116). TREM2 forms a receptor-signaling complex with TYROBP, which mediates signaling and cell activation following ligand binding. Thus, it may be suitable to target TREM2 or neutrophils receptor (e.g. Ly-6G, CD16) on myeloid cells infiltrating MSI-H tumors following ICB resistance. Overall, ICB combination (CTLA4, PD-L1 and others) efficiency may act together with the inhibition of myeloid immunosuppressive programs.

### Vaccination

We and others have characterized MMRd associated recurrent frameshift mutations that generate shared immunogenic neoantigens (28). These frameshifts might serve as targets for cancer vaccine designs (27). A frameshift neoantigen vaccine elicited protective immunity with reduced TMB and improved overall survival in a Lynch Syndrome mouse model (117). This study identified 4 shared immunogenic frameshift peptide neoantigens (Nacad [FSP-1], Maz [FSP-1], Senp6 [FSP-1], Xirp1 [FSP-1]) that induced CD4 and CD8 T cell responses in mice. Using the VCMsh2 mice, conditionally deleted for the DNA repair Msh2 gene in the intestinal tract and developing intestinal cancer, they showed that vaccination with these 4 neoantigens increased adaptive immunity, reduced the intestinal tumor burden, resulting in prolonged overall survival. Better results were obtained using a combination with naproxen treatment, a propionic acid NSAID derivative that has shown pronounced cancer-preventive activity in LS mouse models and increased immune surveillance in LS patients (118, 119). Human trials with frameshift peptides in MSI-H cancers are ongoing (NCT05078866) (120) and other approaches using viral vector platforms are under investigation (121). A nanoparticle vaccine combining neoantigen peptides and a Toll-like receptor 7/8 agonist induced effective anti-tumor activity in combination with ICB in a CRC murine model (122). This suggests that we may use the neoantigen vaccines to prevent MSI-H cancer progression in humans using a similar approach.

In MMRd tumors, aberrant DNA fragments are recognized by cGAS-STING as “non-self” inducing Type I interferon and inflammatory NFkB responses (27). It was shown that cGAS-STING driven type I interferon signaling is required for CXCL10/CCL5-dependent T cell recruitment to MMRd tumor (123, 124). Thus, the use of STING agonists with antigen-specific vaccination strategies may be useful to help to reduce resistance following ICB. However, persistent IFN signaling leads to T cell exhaustion and unfavorable ICB responses (47, 88–90), and more studies would be needed to investigate which patient subsets (early or advanced tumor, with or without combination with ICB) may benefit from this strategy. The use of first-generation intratumoral cyclic dinucleotides was safe but demonstrated only modest systemic activity (125) (111), therefore additional approaches to target this pathway will need to be considered. Finally, in addition to simple antigen-based vaccinations, vaccination strategies that aim to increase the efficiency of antigen-specific immune induction, APC activation and reduce adverse reactions are also being developed in combination with ICB (126). Vaccination approaches should involve strategies that activate DCs (127–130). A phase II trial (NCT02129075) showed that poly-ICLC and fms-like tyrosine kinase 3 (Flt3) ligand pre-treatment enhanced responses to dendritic cell (DC)-targeting vaccines in melanoma patients (131). An mRNA vaccine was shown to induce neoantigen-specific T cell immunity in patients with gastrointestinal cancer (132). As mRNA vaccines are showing efficacy with ICB in tumors with high TMB, these approaches should also be investigated in MSI-H cancers that express high levels of shared neoantigens, potentially leading to the generation of off-the-shelf vaccines (27, 28).

## Conclusion and perspectives

Based on observations from both preclinical MMRd models and clinical cohorts, we hypothesize that the immune resistance observed in MSI-H tumors following ICB may involve the outgrowth of clones with reduced neoantigen presentation, the expression of multiple tumor-associated checkpoint molecules/T cell exhaustion, and coordinated inflammatory hubs of myeloid and stromal immunosuppressive populations in the TME. *In vivo* studies using clinical data and mouse models are key to elucidate these resistance mechanisms, together with new MSI-H CRC patient-derived xenograft models (133), spheroids (134) and *ex vivo* tumor fragment platforms (135). It is crucial to better elucidate these resistance mechanisms of MSI-H tumors during their evolution and following ICB to improve current therapies and immunoprevention strategies.

## Author contributions

All authors listed have made a substantial, direct, and intellectual contribution to the work and approved it for publication.

## References

[1] Li G-M. Mechanisms and functions of DNA mismatch repair. Cell Res (2008) 18:85–98. doi: 10.1038/cr.2007.115 18157157

[2] Karahan B Argon A Yıldırım M Vardar E. Relationship between MLH-1, MSH-2, PMS-2,MSH-6 expression and clinicopathological features in colorectal cancer. Int J Clin Exp Pathol (2015) 8:4044–53. PMC4466979 26097592

[3] Gryfe R Kim H Hsieh ET Aronson MD Holowaty EJ Bull SB. Tumor microsatellite instability and clinical outcome in young patients with colorectal cancer. N Engl J Med (2000) 342:69–77. doi: 10.1056/NEJM200001133420201 10631274

[4] Cancer Genome Atlas Network. Comprehensive molecular characterization of human colon and rectal cancer. Nature (2012) 487:330–7. doi: 10.1038/nature11252 PMC3401966 22810696

[5] Yamamoto H Perez-Piteira J Yoshida T Terada M Itoh F Imai K. Gastric cancers of the microsatellite mutator phenotype display characteristic genetic and clinical features. Gastroenterology (1999) 116:1348–57. doi: 10.1016/s0016-5085(99)70499-3 10348818

[6] Sunakawa Y Lenz H-J. Molecular classification of gastric adenocarcinoma: translating new insights from the cancer genome atlas research network. Curr Treat Options Oncol (2015) 16:17. doi: 10.1007/s11864-015-0331-y 25813036

[7] Cristescu R Lee J Nebozhyn M Kim K-M Ting JC Wong SS. Molecular analysis of gastric cancer identifies subtypes associated with distinct clinical outcomes. Nat Med (2015) 21:449–56. doi: 10.1038/nm.3850 25894828

[8] Diaz-Padilla I Romero N Amir E Matias-Guiu X Vilar E Muggia F. Mismatch repair status and clinical outcome in endometrial cancer: a systematic review and meta-analysis. Crit Rev Oncol Hematol (2013) 88:154–67. doi: 10.1016/j.critrevonc.2013.03.002 23562498

[9] Broaddus RR Lynch HT Chen L-M Daniels MS Conrad P Munsell MF. Pathologic features of endometrial carcinoma associated with HNPCC: a comparison with sporadic endometrial carcinoma. Cancer (2006) 106:87–94. doi: 10.1002/cncr.21560 16323174

[10] Cancer Genome Atlas Research Network Kandoth C Schultz N Cherniack AD Akbani R Liu Y. Integrated genomic characterization of endometrial carcinoma. Nature (2013) 497:67–73. doi: 10.1038/nature12113 23636398 PMC3704730

[11] Billingsley CC Cohn DE Mutch DG Stephens JA Suarez AA Goodfellow PJ. Polymerase ε (POLE) mutations in endometrial cancer: clinical outcomes and implications for lynch syndrome testing. Cancer (2015) 121:386–94. doi: 10.1002/cncr.29046 PMC4304930 25224212

[12] Pal T Permuth-Wey J Kumar A Sellers TA. Systematic review and meta-analysis of ovarian cancers: estimation of microsatellite-high frequency and characterization of mismatch repair deficient tumor histology. Clin Cancer Res Off J Am Assoc Cancer Res (2008) 14:6847–54. doi: 10.1158/1078-0432.CCR-08-1387 PMC2655731 18980979

[13] Xiao X Melton DW Gourley C. Mismatch repair deficiency in ovarian cancer – molecular characteristics and clinical implications. Gynecol Oncol (2014) 132:506–12. doi: 10.1016/j.ygyno.2013.12.003 24333356

[14] Bonneville R Krook MA Kautto EA Miya J Wing MR Chen H-Z. Landscape of microsatellite instability across 39 cancer types. JCO Precis Oncol (2017) 1:1–15. doi: 10.1200/PO.17.00073 PMC5972025 29850653

[15] Ratti M Lampis A Hahne JC Passalacqua R Valeri N. Microsatellite instability in gastric cancer: molecular bases, clinical perspectives, and new treatment approaches. Cell Mol Life Sci (2018) 75:4151–62. doi: 10.1007/s00018-018-2906-9 PMC6182336 30173350

[16] Noh JJ Kim MK Choi MC Lee J-W Park H Jung SG. Frequency of mismatch repair Deficiency/High microsatellite instability and its role as a predictive biomarker of response to immune checkpoint inhibitors in gynecologic cancers. Cancer Res Treat Off J Korean Cancer Assoc (2022) 54:1200–8. doi: 10.4143/crt.2021.828 PMC9582475 34902958

[17] Salem ME Weinberg BA Xiu J El-Deiry WS Hwang JJ Gatalica Z. Comparative molecular analyses of left-sided colon, right-sided colon, and rectal cancers. Oncotarget (2017) 8:86356–68. doi: 10.18632/oncotarget.21169 PMC5689690 29156800

[18] Leskela S Romero I Cristobal E Pérez-Mies B Rosa-Rosa JM Gutierrez-Pecharroman A. Mismatch repair deficiency in ovarian carcinoma: frequency, causes, and consequences. Am J Surg Pathol (2020) 44:649–56. doi: 10.1097/PAS.0000000000001432 32294063

[19] Yen Y-T Chien M Wu P-Y Ho C-C Ho C-T Huang KC-Y. Protein phosphatase 2A inactivation induces microsatellite instability, neoantigen production and immune response. Nat Commun (2021) 12:7297. doi: 10.1038/s41467-021-27620-x 34911954 PMC8674339

[20] Lee V Murphy A Le DT Diaz LA. Mismatch repair deficiency and response to immune checkpoint blockade. Oncol (2016) 21:1200–11. doi: 10.1634/theoncologist.2016-0046 PMC5061538 27412392

[21] Aaltonen LA Peltomäki P Leach FS Sistonen P Pylkkänen L Mecklin JP. Clues to the pathogenesis of familial colorectal cancer. Science (1993) 260:812–6. doi: 10.1126/science.8484121 8484121

[22] Ionov Y Peinado MA Malkhosyan S Shibata D Perucho M. Ubiquitous somatic mutations in simple repeated sequences reveal a new mechanism for colonic carcinogenesis. Nature (1993) 363:558–61. doi: 10.1038/363558a0 8505985

[23] Peltomäki P Aaltonen LA Sistonen P Pylkkänen L Mecklin JP Järvinen H. Genetic mapping of a locus predisposing to human colorectal cancer. Science (1993) 260:810–2. doi: 10.1126/science.8484120 8484120

[24] Thibodeau SN Bren G Schaid D. Microsatellite instability in cancer of the proximal colon. Science (1993) 260:816–9. doi: 10.1126/science.8484122 8484122

[25] Boland CR Goel A. Microsatellite instability in colorectal cancer. Gastroenterology (2010) 138:2073–2087.e3. doi: 10.1053/j.gastro.2009.12.064 20420947 PMC3037515

[26] Woods MO Williams P Careen A Edwards L Bartlett S McLaughlin JR. A new variant database for mismatch repair genes associated with lynch syndrome. Hum Mutat (2007) 28:669–73. doi: 10.1002/humu.20502 17347989

[27] Roudko V Cimen Bozkus C Greenbaum B Lucas A Samstein R Bhardwaj N. Lynch syndrome and MSI-h cancers: from mechanisms to “Off-The-Shelf” cancer vaccines. Front Immunol (2021) 12:757804. doi: 10.3389/fimmu.2021.757804 34630437 PMC8498209

[28] Roudko V Bozkus CC Orfanelli T McClain CB Carr C O’Donnell T. Shared immunogenic poly-epitope frameshift mutations in microsatellite unstable tumors. Cell (2020) 183:1634–1649.e17. doi: 10.1016/j.cell.2020.11.004 33259803 PMC8025604

[29] Rousseau B Foote MB Maron SB Diplas BH Lu S Argilés G. The spectrum of benefit from checkpoint blockade in hypermutated tumors. N Engl J Med (2021) 384:1168–70. doi: 10.1056/NEJMc2031965 PMC8403269 33761214

[30] Diaz LA Shiu K-K Kim T-W Jensen BV Jensen LH Punt C. Pembrolizumab versus chemotherapy for microsatellite instability-high or mismatch repair-deficient metastatic colorectal cancer (KEYNOTE-177): final analysis of a randomised, open-label, phase 3 study. Lancet Oncol (2022) 23:659–70. doi: 10.1016/S1470-2045(22)00197-8 PMC9533375 35427471

[31] Lee BCH Robinson PS Coorens THH Yan HHN Olafsson S Lee-Six H. Mutational landscape of normal epithelial cells in lynch syndrome patients. Nat Commun (2022) 13:2710. doi: 10.1038/s41467-022-29920-2 35581206 PMC9114395

[32] Lemery S Keegan P Pazdur R. First FDA approval agnostic of cancer site [[/amp]]mdash; when a biomarker defines the indication. N Engl J Med (2017) 377:1409–12. doi: 10.1056/NEJMp1709968 29020592

[33] Le DT Uram JN Wang H Bartlett BR Kemberling H Eyring AD. PD-1 blockade in tumors with mismatch-repair deficiency. N Engl J Med (2015) 372:2509–20. doi: 10.1056/NEJMoa1500596 PMC4481136 26028255

[34] Le DT Durham JN Smith KN Wang H Bartlett BR Aulakh LK. Mismatch repair deficiency predicts response of solid tumors to PD-1 blockade. Science (2017) 357:409–13. doi: 10.1126/science.aan6733 PMC5576142 28596308

[35] André T Shiu K-K Kim TW Jensen BV Jensen LH Punt C. Pembrolizumab in Microsatellite-Instability–high advanced colorectal cancer. N Engl J Med (2020) 383:2207–18. doi: 10.1056/NEJMoa2017699 33264544

[36] Ou L Zhang A Cheng Y Chen Y. The cGAS-STING pathway: a promising immunotherapy target. Front Immunol (2021) 12:795048. doi: 10.3389/fimmu.2021.795048 34956229 PMC8695770

[37] Overman MJ Lonardi S Wong KYM Lenz H-J Gelsomino F Aglietta M. Durable clinical benefit with nivolumab plus ipilimumab in DNA mismatch repair–Deficient/Microsatellite instability–high metastatic colorectal cancer. J Clin Oncol (2018) 36:773–9. doi: 10.1200/JCO.2017.76.9901 29355075

[38] André T Cohen R Salem ME. Immune checkpoint blockade therapy in patients with colorectal cancer harboring microsatellite Instability/Mismatch repair deficiency in 2022. Am Soc Clin Oncol Educ Book (2022) 42:233–41. doi: 10.1200/EDBK_349557 35471834

[39] Dantoing E Piton N Salaün M Thiberville L Guisier F. Anti-PD1/PD-L1 immunotherapy for non-small cell lung cancer with actionable oncogenic driver mutations. Int J Mol Sci (2021) 22:6288. doi: 10.3390/ijms22126288 34208111 PMC8230861

[40] da Silva IP Ahmed T Reijers ILM Weppler AM Warner AB Patrinely JR. Ipilimumab alone or ipilimumab plus anti-PD-1 therapy in patients with metastatic melanoma resistant to anti-PD-(L)1 monotherapy: a multicentre, retrospective, cohort study. Lancet Oncol (2021) 22:836–47. doi: 10.1016/S1470-2045(21)00097-8 33989557

[41] Reck M Rodríguez–Abreu D Robinson AG Hui R Csőszi T Fülöp A. Updated analysis of KEYNOTE-024: pembrolizumab versus platinum-based chemotherapy for advanced non–Small-Cell lung cancer with PD-L1 tumor proportion score of 50% or greater. J Clin Oncol (2019) 37:537–46. doi: 10.1200/JCO.18.00149 30620668

[42] Abdullaev S André T Lei M Lenz H-J Novotny J Paulson AS. A phase III study of nivolumab (NIVO), NIVO + ipilimumab (IPI), or chemotherapy (CT) for microsatellite instability-high (MSI-h)/mismatch repair-deficient (dMMR) metastatic colorectal cancer (mCRC): checkmate 8HW. J Clin Oncol (2020) 38:TPS266–6. doi: 10.1200/JCO.2020.38.4_suppl.TPS266

[43] Hu H Kang L Zhang J Wu Z Wang H Huang M. Neoadjuvant PD-1 blockade with toripalimab, with or without celecoxib, in mismatch repair-deficient or microsatellite instability-high, locally advanced, colorectal cancer (PICC): a single-centre, parallel-group, non-comparative, randomised, phase 2 trial. Lancet Gastroenterol Hepatol (2022) 7:38–48. doi: 10.1016/S2468-1253(21)00348-4 34688374

[44] Cercek A Lumish M Sinopoli J Weiss J Shia J Lamendola-Essel M. PD-1 blockade in mismatch repair–deficient, locally advanced rectal cancer. N Engl J Med (2022) 386:2363–76. doi: 10.1056/NEJMoa2201445 PMC9492301 35660797

[45] Chen G Jin Y Guan W-L Zhang R-X Xiao W-W Cai P-Q. Neoadjuvant PD-1 blockade with sintilimab in mismatch-repair deficient, locally advanced rectal cancer: an open-label, single-centre phase 2 study. Lancet Gastroenterol Hepatol (2023) 8:422–31. doi: 10.1016/S2468-1253(22)00439-3 36870360

[46] Kim JH Seo M-K Lee JA Yoo S-Y Oh HJ Kang H. Genomic and transcriptomic characterization of heterogeneous immune subgroups of microsatellite instability-high colorectal cancers. J Immunother Cancer (2021) 9:e003414. doi: 10.1136/jitc-2021-003414 34903553 PMC8672019

[47] Pelka K Hofree M Chen JH Sarkizova S Pirl JD Jorgji V. Spatially organized multicellular immune hubs in human colorectal cancer. Cell (2021) 184:4734–4752.e20. doi: 10.1016/j.cell.2021.08.003 34450029 PMC8772395

[48] Chang K Taggart MW Reyes-Uribe L Borras E Riquelme E Barnett RM. Immune profiling of premalignant lesions in patients with lynch syndrome. JAMA Oncol (2018) 4:1085–92. doi: 10.1001/jamaoncol.2018.1482 PMC6087485 29710228

[49] Bohaumilitzky L Kluck K Hüneburg R Gallon R Nattermann J Kirchner M. The different immune profiles of normal colonic mucosa in cancer-free lynch syndrome carriers and lynch syndrome colorectal cancer patients. Gastroenterology (2021) 162:907–19.e10. doi: 10.1053/j.gastro.2021.11.029 34863788

[50] Brand RE Dudley B Karloski E Das R Fuhrer K Pai RK. Detection of DNA mismatch repair deficient crypts in random colonoscopic biopsies identifies lynch syndrome patients. Fam Cancer (2020) 19:169–75. doi: 10.1007/s10689-020-00161-w 31997046

[51] Daniel B Yost KE Hsiung S Sandor K Xia Y Qi Y. Divergent clonal differentiation trajectories of T cell exhaustion. Nat Immunol (2022) 23:1614–27. doi: 10.1038/s41590-022-01337-5 PMC11225711 36289450

[52] Chaudhary B Elkord E. Regulatory T cells in the tumor microenvironment and cancer progression: role and therapeutic targeting. Vaccines (2016) 4:28. doi: 10.3390/vaccines4030028 27509527 PMC5041022

[53] Cheng P Corzo CA Luetteke N Yu B Nagaraj S Bui MM. Inhibition of dendritic cell differentiation and accumulation of myeloid-derived suppressor cells in cancer is regulated by S100A9 protein. J Exp Med (2008) 205:2235–49. doi: 10.1084/jem.20080132 PMC2556797 18809714

[54] Chen X Eksioglu EA Zhou J Zhang L Djeu J Fortenbery N. Induction of myelodysplasia by myeloid-derived suppressor cells. J Clin Invest (2013) 123:4595–611. doi: 10.1172/JCI67580 PMC3809779 24216507

[55] Strauss L Mahmoud MAA Weaver JD Tijaro-Ovalle NM Christofides A Wang Q. Targeted deletion of PD-1 in myeloid cells induces antitumor immunity. Sci Immunol (2020) 5:eaay1863. doi: 10.1126/sciimmunol.aay1863 31901074 PMC7183328

[56] Wang L Sfakianos JP Beaumont KG Akturk G Horowitz A Sebra RP. Myeloid cell-associated resistance to PD-1/PD-L1 blockade in urothelial cancer revealed through bulk and single-cell RNA sequencing. Clin Cancer Res Off J Am Assoc Cancer Res (2021) 27:4287–300. doi: 10.1158/1078-0432.CCR-20-4574 PMC8338756 33837006

[57] Meyer C Cagnon L Costa-Nunes CM Baumgaertner P Montandon N Leyvraz L. Frequencies of circulating MDSC correlate with clinical outcome of melanoma patients treated with ipilimumab. Cancer Immunol Immunother (2014) 63:247–57. doi: 10.1007/s00262-013-1508-5 PMC11029062 24357148

[58] Ruffell B Coussens LM. Macrophages and therapeutic resistance in cancer. Cancer Cell (2015) 27:462–72. doi: 10.1016/j.ccell.2015.02.015 PMC4400235 25858805

[59] Zhu Y Knolhoff BL Meyer MA Nywening TM West BL Luo J. CSF1/CSF1R blockade reprograms tumor-infiltrating macrophages and improves response to T-cell checkpoint immunotherapy in pancreatic cancer models. Cancer Res (2014) 74:5057–69. doi: 10.1158/0008-5472.CAN-13-3723 PMC4182950 25082815

[60] Deryugina EI Quigley JP. Tumor angiogenesis: MMP-mediated induction of intravasation- and metastasis-sustaining neovasculature. Matrix Biol (2015) 44–46:94–112. doi: 10.1016/j.matbio.2015.04.004 PMC5079283 25912949

[61] Zhang J Li S Zhao Y Ma P Cao Y Liu C. Cancer-associated fibroblasts promote the migration and invasion of gastric cancer cells *via* activating IL-17a/JAK2/STAT3 signaling. Ann Transl Med (2020) 8:877. doi: 10.21037/atm-20-4843 32793721 PMC7396760

[62] Niu B Ye K Zhang Q Lu C Xie M McLellan MD. MSIsensor: microsatellite instability detection using paired tumor-normal sequence data. Bioinformatics (2014) 30:1015–6. doi: 10.1093/bioinformatics/btt755 PMC3967115 24371154

[63] Mandal R Samstein RM Lee K-W Havel JJ Wang H Krishna C. Genetic diversity of tumors with mismatch repair deficiency influences anti-PD-1 immunotherapy response. Science (2019) 364:485–91. doi: 10.1126/science.aau0447 PMC6685207 31048490

[64] Samstein RM Lee C-H Shoushtari AN Hellmann MD Shen R Janjigian YY. Tumor mutational load predicts survival after immunotherapy across multiple cancer types. Nat Genet (2019) 51:202–6. doi: 10.1038/s41588-018-0312-8 PMC6365097 30643254

[65] Das A Sudhaman S Morgenstern D Coblentz A Chung J Stone SC. Genomic predictors of response to PD-1 inhibition in children with germline DNA replication repair deficiency. Nat Med (2022) 28:1–11. doi: 10.1038/s41591-021-01581-6 34992263 PMC8799468

[66] Bellone S Roque DM Siegel ER Buza N Hui P Bonazzoli E. A phase 2 evaluation of pembrolizumab for recurrent lynch-like versus sporadic endometrial cancers with microsatellite instability. Cancer (2021) 28:1206–18. doi: 10.1002/cncr.34025 PMC9465822 34875107

[67] Chow RD Michaels T Bellone S Hartwich TMP Bonazzoli E Iwasaki A. Distinct mechanisms of mismatch repair deficiency delineate two modes of response to PD-1 immunotherapy in endometrial carcinoma. Cancer Discov (2022) 13:CD–22-0686. doi: 10.1158/2159-8290.CD-22-0686 PMC9905265 36301137

[68] Shklovskaya E Lee JH Lim SY Stewart A Pedersen B Ferguson P. Tumor MHC expression guides first-line immunotherapy selection in melanoma. Cancers (2020) 12:3374. doi: 10.3390/cancers12113374 33202676 PMC7696726

[69] Grasso CS Giannakis M Wells DK Hamada T Mu XJ Quist M. Genetic mechanisms of immune evasion in colorectal cancer. Cancer Discovery (2018) 8:730–49. doi: 10.1158/2159-8290.CD-17-1327 PMC5984687 29510987

[70] Middha S Yaeger R Shia J Stadler ZK King S Guercio S. Majority of B2M-mutant and -deficient colorectal carcinomas achieve clinical benefit from immune checkpoint inhibitor therapy and are microsatellite instability-high. JCO Precis Oncol (2019) 3:1–14. doi: 10.1200/PO.18.00321 PMC6469719 31008436

[71] de Vries NL van de Haar J Veninga V Chalabi M Ijsselsteijn ME van der Ploeg M. γδ T cells are effectors of immunotherapy in cancers with HLA class I defects. Nature (2023) 613:1–8. doi: 10.1038/s41586-022-05593-1 PMC9876799 36631610

[72] Germano G Lu S Rospo G Lamba S Rousseau B Fanelli S. CD4 T cell-dependent rejection of beta-2 microglobulin null mismatch repair-deficient tumors. Cancer Discov (2021) 11:1844–59. doi: 10.1158/2159-8290.CD-20-0987 33653693

[73] Rooney MS Shukla SA Wu CJ Getz G Hacohen N. Molecular and genetic properties of tumors associated with local immune cytolytic activity. Cell (2015) 160:48–61. doi: 10.1016/j.cell.2014.12.033 25594174 PMC4856474

[74] Gao Z Tao Y Lai Y Wang Q Li Z Peng S. Immune cytolytic activity as an indicator of immune checkpoint inhibitors treatment for prostate cancer. Front Bioeng Biotechnol (2020) 8:930. doi: 10.3389/fbioe.2020.00930 32850758 PMC7423880

[75] Oshi M Kawaguchi T Yan L Peng X Qi Q Tian W. Immune cytolytic activity is associated with reduced intra-tumoral genetic heterogeneity and with better clinical outcomes in triple negative breast cancer. Am J Cancer Res (2021) 11:3628–44. PMC8332854 34354864

[76] Chen Q Wang C Lei X Huang T Zhou R Lu Y. Immune cytolytic activity for comprehensive insights of the immune landscape in endometrial carcinoma. J Oncol (2022) 2022:9060243. doi: 10.1155/2022/9060243 35898926 PMC9313908

[77] Van Allen EM Miao D Schilling B Shukla SA Blank C Zimmer L. Genomic correlates of response to CTLA-4 blockade in metastatic melanoma. Science (2015) 350:207–11. doi: 10.1126/science.aad0095 PMC5054517 26359337

[78] Narayanan S Kawaguchi T Yan L Peng X Qi Q Takabe K. Cytolytic activity score to assess anticancer immunity in colorectal cancer. Ann Surg Oncol (2018) 25:2323–31. doi: 10.1245/s10434-018-6506-6 PMC6237091 29770915

[79] Palomero J Panisello C Lozano-Rabella M Tirtakasuma R Díaz-Gómez J Grases D. Biomarkers of tumor-reactive CD4+ and CD8+ TILs associate with improved prognosis in endometrial cancer. J Immunother Cancer (2022) 10:e005443. doi: 10.1136/jitc-2022-005443 36581331 PMC9806064

[80] Piulats JM Guerra E Gil-Martín M Roman-Canal B Gatius S Sanz-Pamplona R. Molecular approaches for classifying endometrial carcinoma. Gynecol Oncol (2017) 145:200–7. doi: 10.1016/j.ygyno.2016.12.015 28040204

[81] Phillips SM Banerjea A Feakins R Li SR Bustin SA Dorudi S. Tumour-infiltrating lymphocytes in colorectal cancer with microsatellite instability are activated and cytotoxic. Br J Surg (2004) 91:469–75. doi: 10.1002/bjs.4472 15048750

[82] Ganesan S Mehnert J. Biomarkers for response to immune checkpoint blockade. Annu Rev Cancer Biol (2020) 4:331–51. doi: 10.1146/annurev-cancerbio-030419-033604

[83] Somasundaram R Connelly T Choi R Choi H Samarkina A Li L. Tumor-infiltrating mast cells are associated with resistance to anti-PD-1 therapy. Nat Commun (2021) 12:346. doi: 10.1038/s41467-020-20600-7 33436641 PMC7804257

[84] Yu Y Blokhuis B Derks Y Kumari S Garssen J Redegeld F. Human mast cells promote colon cancer growth *via* bidirectional crosstalk: studies in 2D and 3D coculture models. Oncoimmunology (2018) 7:e1504729. doi: 10.1080/2162402X.2018.1504729 30377568 PMC6205014

[85] Ayers M Lunceford J Nebozhyn M Murphy E Loboda A Kaufman DR. IFN-γ–related mRNA profile predicts clinical response to PD-1 blockade. J Clin Invest (2017) 127:2930–40. doi: 10.1172/JCI91190 PMC5531419 28650338

[86] Litchfield K Reading JL Puttick C Thakkar K Abbosh C Bentham R. Meta-analysis of tumor- and T cell-intrinsic mechanisms of sensitization to checkpoint inhibition. Cell (2021) 184:596–614.e14. doi: 10.1016/j.cell.2021.01.002 33508232 PMC7933824

[87] Cristescu R Mogg R Ayers M Albright A Murphy E Yearley J. Pan-tumor genomic biomarkers for PD-1 checkpoint blockade–based immunotherapy. Science (2018) 362:eaar3593. doi: 10.1126/science.aar3593 30309915 PMC6718162

[88] Boukhaled GM Gadalla R Elsaesser HJ Abd-Rabbo D Quevedo R Yang SYC. Pre-encoded responsiveness to type I interferon in the peripheral immune system defines outcome of PD1 blockade therapy. Nat Immunol (2022) 23:1273–83. doi: 10.1038/s41590-022-01262-7 35835962

[89] Dubrot J Du PP Lane-Reticker SK Kessler EA Muscato AJ Mehta A. *In vivo* CRISPR screens reveal the landscape of immune evasion pathways across cancer. Nat Immunol (2022) 23:1495–506. doi: 10.1038/s41590-022-01315-x 36151395

[90] Sui Q Zhang X Chen C Tang J Yu J Li W. Inflammation promotes resistance to immune checkpoint inhibitors in high microsatellite instability colorectal cancer. Nat Commun (2022) 13:7316. doi: 10.1038/s41467-022-35096-6 36443332 PMC9705377

[91] Chida K Kawazoe A Suzuki T Kawazu M Ueno T Takenouchi K. Transcriptomic profiling of MSI-H/dMMR gastrointestinal tumors to identify determinants of responsiveness to anti-PD-1 therapy. Clin Cancer Res Off J Am Assoc Cancer Res (2022) 28:0041. doi: 10.1158/1078-0432.CCR-22-0041 PMC9365358 35254400

[92] Amodio V Mauri G Reilly NM Sartore-Bianchi A Siena S Bardelli A. Mechanisms of immune escape and resistance to checkpoint inhibitor therapies in mismatch repair deficient metastatic colorectal cancers. Cancers (2021) 13:2638. doi: 10.3390/cancers13112638 34072037 PMC8199207

[93] Kalbasi A Ribas A. Tumour-intrinsic resistance to immune checkpoint blockade. Nat Rev Immunol (2020) 20:25–39. doi: 10.1038/s41577-019-0218-4 31570880 PMC8499690

[94] Mestrallet G Sone K Bhardwaj N. Strategies to overcome DC dysregulation in the tumor microenvironment. Front Immunol (2022) 13:980709. doi: 10.3389/fimmu.2022.980709 36275666 PMC9583271

[95] Sakuishi K Apetoh L Sullivan JM Blazar BR Kuchroo VK Anderson AC. Targeting Tim-3 and PD-1 pathways to reverse T cell exhaustion and restore anti-tumor immunity. J Exp Med (2010) 207:2187–94. doi: 10.1084/jem.20100643 PMC2947065 20819927

[96] Rangachari M Zhu C Sakuishi K Xiao S Karman J Chen A. Bat3 promotes T cell responses and autoimmunity by repressing Tim-3–mediated cell death and exhaustion. Nat Med (2012) 18:1394–400. doi: 10.1038/nm.2871 PMC3491118 22863785

[97] Dixon KO Tabaka M Schramm MA Xiao S Tang R Dionne D. TIM-3 restrains anti-tumour immunity by regulating inflammasome activation. Nature (2021) 595:101–6. doi: 10.1038/s41586-021-03626-9 PMC8627694 34108686

[98] Dixon KO Schorer M Nevin J Etminan Y Amoozgar Z Kondo T. Functional anti-TIGIT antibodies regulate development of autoimmunity and antitumor immunity. J Immunol Baltim Md 1950 (2018) 200:3000–7. doi: 10.4049/jimmunol.1700407 PMC5893394 29500245

[99] Han J-H Cai M Grein J Perera S Wang H Bigler M. Effective anti-tumor response by TIGIT blockade associated with FcγR engagement and myeloid cell activation. Front Immunol (2020) 11:573405. doi: 10.3389/fimmu.2020.573405 33117369 PMC7577118

[100] Nebot-Bral L Hollebecque A Yurchenko AA de Forceville L Danjou M Jouniaux J-M. Overcoming resistance to αPD-1 of MMR-deficient tumors with high tumor-induced neutrophils levels by combination of αCTLA-4 and αPD-1 blockers. J Immunother Cancer (2022) 10:e005059. doi: 10.1136/jitc-2022-005059 35896284 PMC9335020

[101] Triebel F Jitsukawa S Baixeras E Roman-Roman S Genevee C Viegas-Pequignot E. LAG-3, a novel lymphocyte activation gene closely related to CD4. J Exp Med (1990) 171:1393–405. doi: 10.1084/jem.171.5.1393 PMC2187904 1692078

[102] Gao J Ward JF Pettaway CA Shi LZ Subudhi SK Vence LM. VISTA is an inhibitory immune checkpoint that is increased after ipilimumab therapy in patients with prostate cancer. Nat Med (2017) 23:551–5. doi: 10.1038/nm.4308 PMC5466900 28346412

[103] Balan S Radford KJ Bhardwaj N. Chapter two - unexplored horizons of cDC1 in immunity and tolerance. In: Alt FW, editor. Advances in immunology Icahn School of Medicine at Mount Sinai, New York City, NY, United States: Academic Press (2020). p. 49–91. doi: 10.1016/bs.ai.2020.10.002 33190733

[104] Mestrallet G Auvré F Schenowitz C Carosella ED LeMaoult J Martin MT. Human keratinocytes inhibit CD4+ T-cell proliferation through TGFB1 secretion and surface expression of HLA-G1 and PD-L1 immune checkpoints. Cells (2021) 10:1438. doi: 10.3390/cells10061438 34201301 PMC8227977

[105] Mestrallet G Carosella ED Martin MT Rouas-Freiss N Fortunel NO LeMaoult J. Immunosuppressive properties of epidermal keratinocytes differ according to their immaturity status. Front Immunol (2022) 13:786859. doi: 10.3389/fimmu.2022.786859 35222373 PMC8878806

[106] Mestrallet G Rouas-Freiss N LeMaoult J Fortunel NO Martin MT. Skin immunity and tolerance: focus on epidermal keratinocytes expressing HLA-G. Front Immunol (2021) 12:772516. doi: 10.3389/fimmu.2021.772516 34938293 PMC8685247

[107] Zeestraten ECM Reimers MS Saadatmand S Dekker J-WT Liefers GJ van den Elsen PJ. Combined analysis of HLA class I, HLA-e and HLA-G predicts prognosis in colon cancer patients. Br J Cancer (2014) 110:459–68. doi: 10.1038/bjc.2013.696 PMC3899753 24196788

[108] Garziera M Bidoli E Cecchin E Mini E Nobili S Lonardi S. HLA-G 3’UTR polymorphisms impact the prognosis of stage II-III CRC patients in fluoropyrimidine-based treatment. PloS One (2015) 10:e0144000. doi: 10.1371/journal.pone.0144000 26633805 PMC4669157

[109] Cao M Yie S-M Liu J Ye SR Xia D Gao E. Plasma soluble HLA-G is a potential biomarker for diagnosis of colorectal, gastric, esophageal and lung cancer. Tissue Antigens (2011) 78:120–8. doi: 10.1111/j.1399-0039.2011.01716.x 21726203

[110] Ozato Y Kojima Y Kobayashi Y Hisamatsu Y Toshima T Yonemura Y. Spatial and single-cell transcriptomics decipher the cellular environment containing HLA-g+ cancer cells and SPP1+ macrophages in colorectal cancer. Cell Rep (2023) 42:111929. doi: 10.1016/j.celrep.2022.111929 36656712

[111] Tsui C Kretschmer L Rapelius S Gabriel SS Chisanga D Knöpper K. MYB orchestrates T cell exhaustion and response to checkpoint inhibition. Nature (2022) 609:1–7. doi: 10.1038/s41586-022-05105-1 PMC9452299 35978192

[112] Barrington DA Calo C Baek J Brown M Wagner V Gonzalez L. Beyond mismatch repair deficiency? pre-treatment neutrophil-to-lymphocyte ratio is associated with improved overall survival in patients with recurrent endometrial cancer treated with immunotherapy. Gynecol Oncol (2022) 166:522–9. doi: 10.1016/j.ygyno.2022.07.010 35907683

[113] Molgora M Esaulova E Vermi W Hou J Chen Y Luo J. TREM2 modulation remodels the tumor myeloid landscape enhancing anti-PD-1 immunotherapy. Cell (2020) 182:886–900.e17. doi: 10.1016/j.cell.2020.07.013 32783918 PMC7485282

[114] Katzenelenbogen Y Sheban F Yalin A Yofe I Svetlichnyy D Jaitin DA. Coupled scRNA-seq and intracellular protein activity reveal an immunosuppressive role of TREM2 in cancer. Cell (2020) 182:872–885.e19. doi: 10.1016/j.cell.2020.06.032 32783915

[115] Park MD Silvin A Ginhoux F Merad M. Macrophages in health and disease. Cell (2022) 185:4259–79. doi: 10.1016/j.cell.2022.10.007 PMC9908006 36368305

[116] Bouchon A Dietrich J Colonna M. Cutting edge: inflammatory responses can be triggered by TREM-1, a novel receptor expressed on neutrophils and Monocytes1. J Immunol (2000) 164:4991–5. doi: 10.4049/jimmunol.164.10.4991 10799849

[117] Gebert J Gelincik O Oezcan-Wahlbrink M Marshall JD Hernandez-Sanchez A Urban K. Recurrent frameshift neoantigen vaccine elicits protective immunity with reduced tumor burden and improved overall survival in a lynch syndrome mouse model. Gastroenterology (2021) 161:1288–1302.e13. doi: 10.1053/j.gastro.2021.06.073 34224739 PMC10184299

[118] Reyes-Uribe L Wu W Gelincik O Bommi PV Francisco-Cruz A Solis LM. Naproxen chemoprevention promotes immune activation in lynch syndrome colorectal mucosa. Gut (2021) 70:555–66. doi: 10.1136/gutjnl-2020-320946 PMC7790993 32641470

[119] Martín-López J Gasparini P Coombes K Croce CM Boivin GP Fishel R. Mutation of TGFβ-RII eliminates NSAID cancer chemoprevention. Oncotarget (2017) 9:12554–61. doi: 10.18632/oncotarget.23792 PMC5849154 29560090

[120] Overman M Fakih M Le D Shields A Pedersen K Shah M. 410 phase I interim study results of nous-209, an off-the-shelf immunotherapy, with pembrolizumab, for the treatment of tumors with a deficiency in mismatch repair/microsatellite instability (dMMR/MSI). J Immunother Cancer (2021) 9. doi: 10.1136/jitc-2021-SITC2021.410

[121] Kloor M Reuschenbach M Pauligk C Karbach J Rafiyan M-R Al-Batran S-E. A frameshift peptide neoantigen-based vaccine for mismatch repair-deficient cancers: a phase I/IIa clinical trial. Clin Cancer Res Off J Am Assoc Cancer Res (2020) 26:4503–10. doi: 10.1158/1078-0432.CCR-19-3517 32540851

[122] Baharom F Ramirez-Valdez RA Tobin KKS Yamane H Dutertre C-A Khalilnezhad A. Intravenous nanoparticle vaccination generates stem-like TCF1+ neoantigen-specific CD8+ T cells. Nat Immunol (2021) 22:41–52. doi: 10.1038/s41590-020-00810-3 33139915 PMC7746638

[123] Mowat C Mosley SR Namdar A Schiller D Baker K. Anti-tumor immunity in mismatch repair-deficient colorectal cancers requires type I IFN–driven CCL5 and CXCL10. J Exp Med (2021) 218:e20210108. doi: 10.1084/jem.20210108 34297038 PMC8313406

[124] Lu C Guan J Lu S Jin Q Rousseau B Lu T. DNA Sensing in mismatch repair-deficient tumor cells is essential for anti-tumor immunity. Cancer Cell (2021) 39:96–108.e6. doi: 10.1016/j.ccell.2020.11.006 33338425 PMC9477183

[125] Amouzegar A Chelvanambi M Filderman JN Storkus WJ Luke JJ. STING agonists as cancer therapeutics. Cancers (2021) 13:2695. doi: 10.3390/cancers13112695 34070756 PMC8198217

[126] Ott PA Hu-Lieskovan S Chmielowski B Govindan R Naing A Bhardwaj N. A phase ib trial of personalized neoantigen therapy plus anti-PD-1 in patients with advanced melanoma, non-small cell lung cancer, or bladder cancer. Cell (2020) 183:347–362.e24. doi: 10.1016/j.cell.2020.08.053 33064988

[127] Saxena M Bhardwaj N. Re-emergence of dendritic cell vaccines for cancer treatment. Trends Cancer (2018) 4:119–37. doi: 10.1016/j.trecan.2017.12.007 PMC5823288 29458962

[128] Roberts EW Broz ML Binnewies M Headley MB Nelson AE Wolf DM. Critical role for CD103(+)/CD141(+) dendritic cells bearing CCR7 for tumor antigen trafficking and priming of T cell immunity in melanoma. Cancer Cell (2016) 30:324–36. doi: 10.1016/j.ccell.2016.06.003 PMC5374862 27424807

[129] Fonteneau J-F Larsson M Bhardwaj N. Dendritic cell–dead cell interactions: implications and relevance for immunotherapy. J Immunother (2001) 24:294–304. doi: 10.1097/00002371-200107000-00005 11565831

[130] Balan S Finnigan J Bhardwaj N. DC Strategies for eliciting mutation-derived tumor antigen responses in patients. Cancer J Sudbury Mass (2017) 23:131–7. doi: 10.1097/PPO.0000000000000251 PMC5520811 28410301

[131] Bhardwaj N Friedlander PA Pavlick AC Ernstoff MS Gastman BR Hanks BA. Flt3 ligand augments immune responses to anti-DEC-205-NY-ESO-1 vaccine through expansion of dendritic cell subsets. Nat Cancer (2020) 1:1204–17. doi: 10.1038/s43018-020-00143-y 35121932

[132] Cafri G Gartner JJ Zaks T Hopson K Levin N Paria BC. mRNA vaccine–induced neoantigen-specific T cell immunity in patients with gastrointestinal cancer. J Clin Invest (2020) 130:5976–88. doi: 10.1172/JCI134915 PMC7598064 33016924

[133] Suto H Funakoshi Y Nagatani Y Imamura Y Toyoda M Kiyota N. Microsatellite instability-high colorectal cancer patient-derived xenograft models for cancer immunity research. J Cancer Res Ther (2021) 17:1358–69. doi: 10.4103/jcrt.JCRT_1092_20 34916366

[134] Lugand L Mestrallet G Laboureur R Dumont C Bouhidel F Djouadou M. Methods for establishing a renal cell carcinoma tumor spheroid model with immune infiltration for immunotherapeutic studies. Front Oncol (2022) 12:898732. doi: 10.3389/fonc.2022.898732 35965544 PMC9366089

[135] Voabil P de Bruijn M Roelofsen LM Hendriks SH Brokamp S van den Braber M. An *ex vivo* tumor fragment platform to dissect response to PD-1 blockade in cancer. Nat Med (2021) 27:1250–61. doi: 10.1038/s41591-021-01398-3 34239134

